# Experimental Study on Profile Control of Polymer and Weak Gel Molecules in Porous Media

**DOI:** 10.3390/gels8080467

**Published:** 2022-07-26

**Authors:** Xuanran Li, Jing Wei, Lun Zhao, Jun Ni, Libing Fu, Jincai Wang

**Affiliations:** 1 Research Institute of Petroleum Exploration and Development (RIPED), Beijing 100083, China; zhaolun@petrochina.com.cn (L.Z.); nijun@petrochina.com.cn (J.N.); fulibing@petrochina.com.cn (L.F.); wangjincai1@petrochina.com.cn (J.W.); 2 Xinjiang Keli New Technology Development Co., Ltd., Karamay 834000, China; weijing@xjkeli.com

**Keywords:** weak gel, porous media shear, molecular coil size, viscoelastic modulus, molecular aggregation morphology

## Abstract

Weak gel is a gel system formed by the mixing and crosslinking of a low-concentration polymer and a slow-release crosslinker. It can be used for profile control in deep reservoir, but its effect is greatly affected by mechanical shearing. Currently, the shearing effect on weak gel is mainly studied by way of mechanical stirring, while the effect of porous media shear on weak gel molecules and properties has been rarely discussed. In this paper, polymer solution, aluminum gel and phenolic gel were prepared. The molecular coil size, viscoelastic modulus and microscopic aggregation morphology in water solution of three systems before and after core shearing were investigated, and the injection performance of the three systems in cores with different permeabilities was tested by physical simulation experiments. The study results show that at equivalent permeability, the system with a larger equivalent sphere diameter of molecular coil is more seriously sheared and suffers greater viscosity loss. In the core with permeability of 1.0 D, polymer solution remains as the aggregation, while phenolic gel and aluminum gel cannot form network aggregations and they are inferior to polymer solution in migration capacity in the mid-deep part of the core. In the core with permeability of 1–5.8 D, the polymer solution remains as a Newtonian fluid, while phenolic gel and aluminum gel become purely viscous non-Newtonian fluids. The elastic modulus of aluminum gel and phenolic gel is four times more than that of a polymer. In the core with permeability higher than 8.5 D, aluminum gel and phenolic gel migrate with less effect by core shearing, and their profile control capacity in deep reservoir is higher than that of the polymer. In the core with permeability lower than 8.5 D, because the monomolecular activity of weak gels becomes poor, they migrate in porous media with more effect by core shearing, and their profile control and oil displacement capacity in deep reservoir is lower than that of the polymer.

## 1. Introduction

As one mechanism of chemical flooding, a high molecular polymer is added to a water solution to increase water viscosity and reduce water-oil mobility ratio, thereby inhibiting the fingering of the displacing phase in the reservoir, so that the water injection profile can be controlled to improve the macroscopic sweep efficiency. Field and experimental studies have indicated that polymer flooding performs well in controlling the water injection profile for reservoirs, with a permeability variation coefficient below 0.75 [1,2,3,4], but less capable for reservoirs with strong heterogeneity, especially those with large pore throats or fractures. Bulk gel is a gel system formed by intermolecular crosslinking of the polymer and crosslinker under certain conditions. It has higher viscoelastic properties owing to the crosslinking between molecules, so it can be used to plug high-permeability layer or fractures in the formation to improve the macroscopic sweep efficiency. However, because its fluidity is poorer than that of a linear high molecular polymer, it can only act on the strata within 10 m from the wellbore if multi-scale pores coexist in the reservoir; thus, its profile control and oil displacement capability is limited. In order to improve the deep profile control and oil displacement capability of gel, a low-concentration polymer and slow-release crosslinker can be mixed and injected into the well to form weak gel in deep formations. Since weak gel has both the migration ability of high molecular polymer and the profile control ability of bulk gel, it has become a hotspot in studies [5,6,7,8,9,10,11].

The gelling effect of weak gel greatly affects its oil displacement and profile control performance. When the polymer water solution is injected, it may degrade easily due to mechanical shearing when flowing through the polymer injection pump, pipeline, static mixer and formations, resulting in a significant loss of polymer viscosity [12,13,14,15,16,17]. Recent studies have shown a similar problem for weak gel. After the partially hydrolyzed polyacrylamide/aluminum citrate gel (hereinafter referred to as aluminum gel) is stirred by a rapid stirrer, the size of the gel coil measured by the light scattering method is significantly reduced, and the faster the stirring speed, the more obvious the reduction in the gel coil size is [10]. Although mechanical stirring can be adopted to simulate gel degradation when flowing through the polymer injection pump and pipeline, it does not work for polymer degradation when flowing through porous media of the formations. In this paper, with the sand-packed core as the porous media of the formation, the molecular coil size, viscoelasticity and microscopic aggregation morphology in the polymer water solution (partially hydrolyzed polyacrylamide or HPAM), polymer/aluminum gel and polymer/phenolic resin gel (hereinafter referred to as phenolic gel) before and after flowing through the sand-packed core were investigated, in order to further characterize the effect of porous media shear on the molecular states and properties of the polymer and weak gel and clarify the migration behaviors and profile control and oil displacement effects of polymer and gel systems in cores with different permeabilities. The study results provide a reference for the design of slug and the selection of profile control and an oil displacement agent in staged profile control and oil displacement in old oilfields with a high water cut.

## 2. Experiment

### 2.1. Experimental Materials and Instruments

Chemicals: The partially hydrolyzed polyacrylamide (HPAM, intrinsic viscosity: ≥1.2 × 10^7^, solid content: 90.0%, degree of hydrolysis: 22.5%) is commonly used as a thickener in Daqing Oilfield, Qinghan Oilfield, Xinjiang Oilfield, and so on. It is a type II polymer for the oil displacement of CNPC; phenolic crosslinker (effective content of 100%); aluminum citrate crosslinker (aluminum crosslinker, effective content of 2.5%); The experimental chemicals were provided by Xinjiang Keli New Technology Development Co., Ltd. located in Karamay City, Xinjiang Autonomous Region, China.

Water: Simulated formation water, with the total salinity of 60,000 mg/L, the Ca^2+^ content of 2800 mg/L, and the Mg^2+^ content of 1000 mg/L.

Crude oil: Crude oil from a single well, with a viscosity of 921 mPa·s at 30 °C; diluted with aviation kerosene to the viscosity of 210 mPa·s at 30 °C formation temperature.

Preparation of weak gel: The phenolic crosslinker (500 mg/L) and the aluminum crosslinker (40:1 for polymer/crosslinker ratio) were added in the polymer solution (concentration of 1500 mg/L), and the mixture was placed in a 30 °C thermostat for 7 days to coagulate and form a weak gel.

Instruments: Ubbelohde viscometer (dilution type), Anton Paar MCR301 rheometer, PLYMPUS CX31, MF-2 microscope, constant-temperature and constant-pressure displacement device, sand-packed core with a length of 8 cm and diameter of 2.5 cm.

### 2.2. Experimental Methods

Test on molecular coil size: The intrinsic viscosity test procedures in the Recommended Practices for Evaluation of Polymers Used in Enhanced Oil Recovery (API RP 63) were followed. Firstly, the Ubbelohde viscometer was used to test the intrinsic viscosity [*η*] of the polymer and weak gel. Then, the viscosity-average molecular weight ${\bar{M}}_{\eta }$ of the polymer and weak gel was calculated by the formula $[\eta ]=K{\bar{M}}_{\eta }{}^{a}$, where K and a are empirical constants, with K = 6.31 × 10^−3^ and a = 0.8 in this study. Finally, the equivalent sphere diameter of molecular coil of polymer and weak gel was determined from the formula $d_{equ,[\eta ]}=1.08\times 10^{-4}{\left({\bar{M}}_{\eta }[\eta ]\right)}^{1/3}\mu m$ [9,10,11,14].

Test on viscoelasticity: Firstly, 1500 mg/L polymer, aluminum gel and phenolic gel were selected. Then, in the linear viscoelastic region (strain of 10%), and given the frequency of 1 Hz, the CP25-21TG-SN2557 laminoplasty system of MCR302 rheometer was used to test the viscoelastic modulus at *d* = 0.103 mm and 30 °C. Thus, the viscoelastic properties of the polymer and weak gel were obtained.

Test on change of microscopic aggregation morphology in water solution: The 1500 mg/L polymer, aluminum gel and phenolic gel were selected to observe the change in solution state before and after shearing under a microscope.

Test on resistance factor and residual resistance factor [18,19]: With the constant-temperature and constant-pressure displacement device and at an injection rate of 1 mL/min, the stable water injection pressure *p*_w_ of the sand-packed core was measured. Then, the polymer or gel was injected, and the stable polymer injection pressure *p_p_* was measured. The resistance factor of the polymer solution was determined by

$$F_{R}(P)={\left(\frac{p_{p}}{p_{w}}\right)}_{q}$$

where, *F_R_*(*P*) is the resistance factor; *p_w_* is the stable water injection pressure, MPa; *p_p_* is the stable polymer injection pressure, MPa.

Then, formation water was injected at the same rate as the polymer injection rate into the core with the polymer injected. When the stable water injection pressure *p_w_*′ was reached, the residual resistance factor of the polymer solution was determined by

$$F_{RR}(P)={\left(\frac{{p^{\prime }}_{W}}{p_{w}}\right)}_{q}$$

where, *F_R__R_*(*P*) is the residual resistance factor; *p_w_*′ is the stable water injection pressure for second time, MPa; *p_w_* is the stable water injection pressure or first time, MPa.

If the pressure continues to rise when the polymer or weak gel is injected, it means that the polymer or weak gel results in blockage in the core.

**Figure 1:**
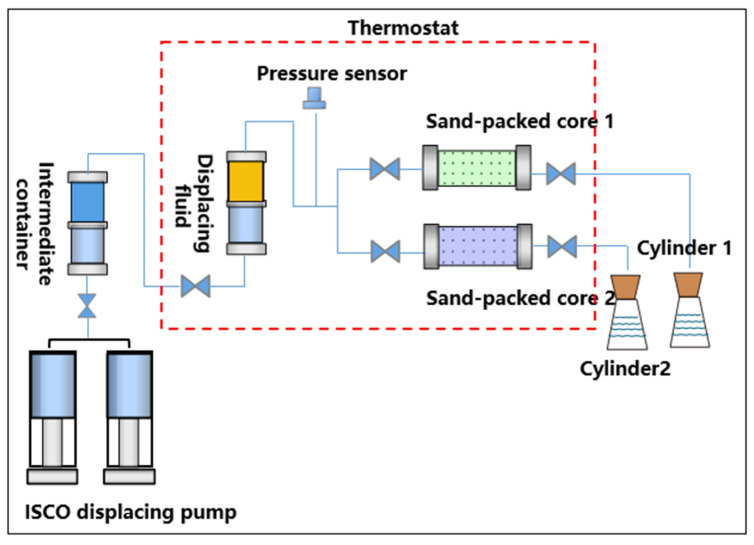
Flow chart of double-tube displacement experiment device.

Test on oil displacement efficiency and fluid diversion ability: With the constant-temperature and constant-pressure displacement device, the double-tube parallel sand-packed core test method was used to simulate the conditions of high and low permeability heterogeneous reservoirs [16,20]. In the experiment, the sand-packed cores were saturated with formation water. Then, the crude oil was used to displace to the bound water saturation, and the two sand-packed cores were connected in parallel. Under the constant temperature of 30 °C and injection rate of 0.2 mL/min, the simulated salt water was used to displace to 98% water cut of produced fluid, and then, 0.3 PV polymer/weak gel was injected. After waiting for coagulation for 7 days, the simulated salt water was used to displace to 98% water cut of produced fluid, and the recovery factor and the percentage of liquid production were calculated (in Figure 1).

## 3. Results and Discussion

### 3.1. Molecular Coil Size of Polymer and Weak Gel before and after Flowing through Sand-Packed Cores

**Figure 2:**
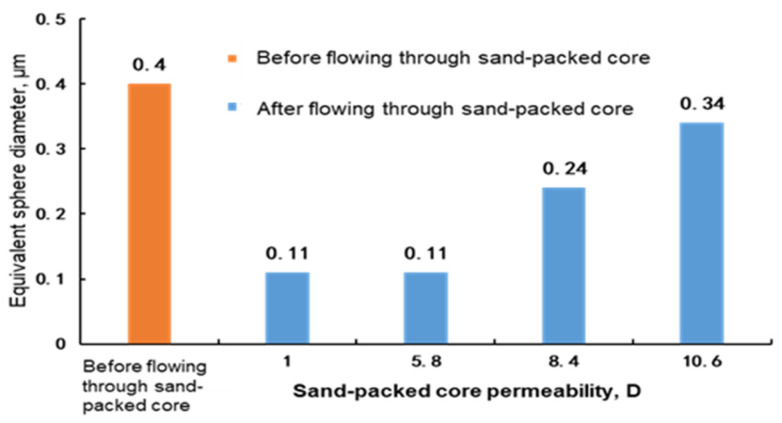
Measurement of equivalent sphere diameter of polymer before and after flowing through sand-packed cores.

**Figure 3:**
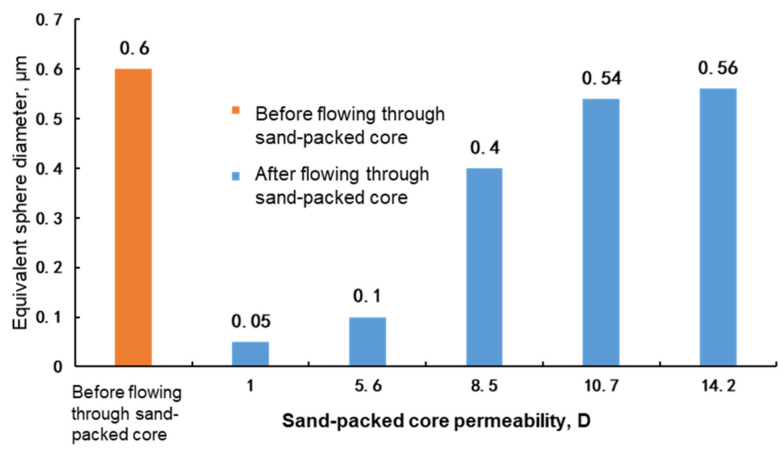
Measurement of equivalent sphere diameter of phenolic gel before and after flowing through sand-packed cores.

**Figure 4:**
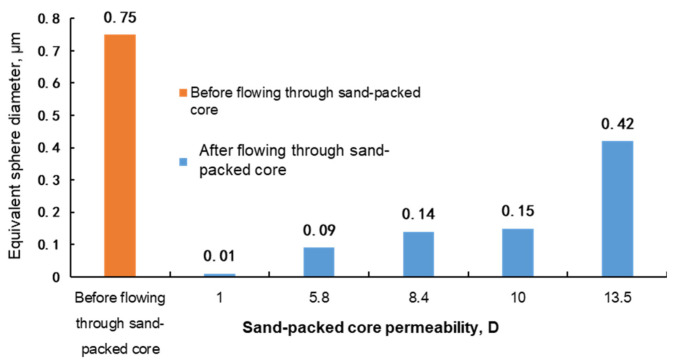
Measurement of equivalent sphere diameter of aluminum gel before and after flowing through sand-packed cores.

The molecular coil size of the polymer and the weak gel were mainly measured with the light scattering method, nuclear pore membrane method and intrinsic viscosity method. The light scattering method measures the molecular coil size by using the high sensitivity nanoparticle analysis, and it is susceptible to the interference of impurities such as dust and requires the high purity of the measured system. The nuclear pore membrane method requires a nuclear pore membrane filter unit; the active force between the polymer and gel molecules is strong, and the molecular size flowing through the nuclear pore membrane is difficult to keep consistent with the sieve size [13,14]. In this paper, the molecular coil size was obtained by the intrinsic viscosity method [11]; that is, considering that polymer and gel molecules disperse in water to become interwound, the molecular coil is equivalent to a sphere to characterize the sizes of different polymer or gel molecules. In the experiment, the sand-packed cores with permeability of 1–15 D were selected to test the molecular coil size, i.e., the equivalent sphere diameter, of the polymer solution and weak gel before and after flowing through the cores. The equivalent sphere diameter was obtained by the experimental method described in Section 2.2. The experimental results are shown in Figure 2, Figure 3 and Figure 4.

It can be seen from Figure 2, Figure 3 and Figure 4 that before flowing through the sand-packed cores, the equivalent sphere diameters of molecular coils of phenolic gel and aluminum gel are 0.60 μm and 0.75 μm, respectively, 50% and 87.5% higher than that (0.40 μm) of polymer. Essentially, the phenolic gel and aluminum gel are weak gels formed by chemical and physical crosslinking of polymer molecules and crosslinkers, and their equivalent sphere diameters of molecular coils are greatly improved owing to the cross linking between molecules.

After flowing through the sand-packed cores, as the core permeability increases, the equivalent sphere diameters of the polymer solution, phenolic gel and aluminum gel gradually decrease, but with slightly different extents and laws.

(1) When the core permeability is less than 5.8 D, the equivalent sphere diameter of the molecular coil of the polymer solution is basically stable. When the core permeability continues to decrease, the molecular coil size is no longer reduced. This indicates that although the polymer solution is sheared in the core, molecules are weakly sheared, because they are not cross linked and have good monomolecular activity, allowing the polymer to easily migrate, and upon a certain extent of shear, the molecular coil size can remain stable. Therefore, under the condition of permeability from 1 D to 10 D, the polymer molecules after shearing can migrate to deep reservoirs to improve the sweep efficiency and oil displacement effect.
(2) When the phenolic gel flows through the core with a permeability higher than 8.5 D, the reduction in the equivalent sphere diameter of the molecular coil is less than 40%, indicating that the molecules of phenolic gel are just weakly cross linked and still have good monomolecular activity, allowing the phenolic gel to easily migrate. Therefore, the phenolic gel has good profile control and oil displacement capability in the deep core with a permeability higher than 8.5 D. However, when the core permeability is reduced to 5.6 D and 1 D, the molecular coil size of phenolic gel is lower than that of polymer solution, indicating that under these permeability conditions, the phenolic gel is greatly sheared and suffers a great viscosity loss, significantly reducing its capability to improve the sweep efficiency in deep reservoir.
(3) The initial equivalent sphere diameter of the molecular coil of the aluminum gel is the largest. However, when flowing through cores with different permeabilities, the absolute value of the equivalent sphere diameter of molecular coil of the aluminum gel is lower than that of the phenolic gel. This indicates that the aluminum gel has weaker monomolecular activity than the phenolic gel, and it is more greatly sheared in cores, so its profile control and oil displacement capability in deep reservoir is weaker than that of the phenolic gel. Similar to the phenolic gel, when the permeability is reduced to 5.6 D and 1 D, the molecular coil size of the aluminum gel is also lower than that of the polymer solution, indicating that under these permeability conditions, the capability of aluminum gel to improve the sweep efficiency in deep reservoir is significantly reduced.

### 3.2. Viscoelasticity of Polymer and Weak Gel before and after Flowing through Sand-Packed Cores

Viscoelasticity has an important influence on the sweep efficiency of the polymer solution. High viscoelastic modulus allows the polymer solution to improve the sweep efficiency in deep formations and adjust the water injection profile. Therefore, the rheometer was used to further test the changes in the viscoelasticity of polymer water solution and weak gels before and after flowing through sand-packed cores [20,21].

**Figure 5:**
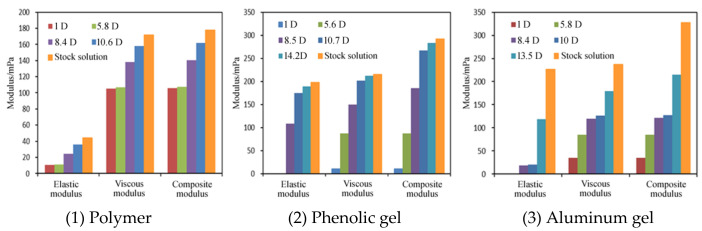
Changes of viscoelastic modulus of three solutions/gels before and after flowing through sand-packed cores with different permeabilities.

It can be seen from Figure 5 that before flowing through sand-packed cores, the viscoelastic modulus of aluminum gel is the highest, followed by phenolic gel and polymer solution in turn. Essentially, for the phenolic gel and aluminum gel formed by the chemical and physical crosslinking of polymer molecules and crosslinkers, the intermolecular interaction force is higher, so their viscoelastic modulus, especially elastic modulus, is greatly improved.

After flowing through sand-packed cores, as the core permeability increases, the viscoelastic moduli of polymer solution, phenolic gel and aluminum gel decrease, but with different extents and laws.

(1) When the core permeability is 10.6 D and 8.4 D, the elastic modulus and viscous modulus of the polymer solution decrease greatly. When the core permeability is less than 5.8 D, the viscoelastic modulus is basically stable. This indicates that the molecular size and aggregation morphology of the polymer solution no longer changes after it encounters a shear force. Ultimately, when the core permeability is 1 D, the elastic modulus is 10.9 MPa, indicating that the polymer remains a non-Newtonian viscoelastic fluid, which can still be capable of improving the sweep efficiency in deep reservoirs by virtue of the viscoelasticity under the condition of low permeability.
(2) When the core permeability is ≥8.5 D, the viscoelastic modulus of the phenolic gel is higher than that of the polymer solution, indicating that under the condition of high permeability, the capability of phenolic gel to improve the sweep efficiency in the formation is higher than that of polymer solution. However, when the core permeability is ≤5.6 D, the elastic modulus is reduced to 0, and the viscous modulus is also significantly reduced to below 100 MPa, making the phenolic gel become a pure viscous non-Newtonian fluid. Therefore, when the core permeability is low, the phenolic gel is less capable than the polymer solution to improve the sweep efficiency in the formation.
(3) When the core permeability is ≥10 D, the viscoelastic modulus of the aluminum gel is significantly higher than that of the polymer solution, indicating that under this condition, the capability of aluminum gel to improve the sweep efficiency in the formation is higher than that of polymer solution. However, when the core permeability is ≤8.4 D, the viscoelastic modulus of aluminum gel is smaller than that of polymer solution, indicating that under this condition, aluminum gel is less capable than polymer solution to improve the sweep efficiency in the formation.

### 3.3. Microscopic Aggregation Morphology in Water Solution of Polymer and Weak Gel before and after Flowing through Sand-Packed Cores

**Figure 6:**
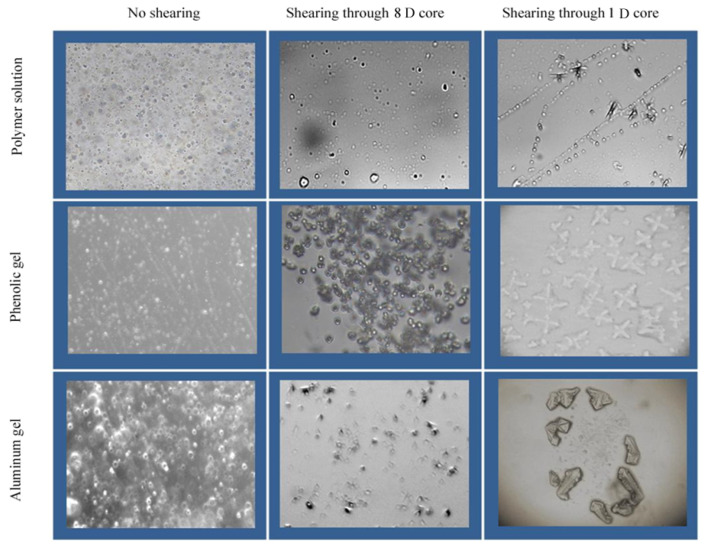
Changes of microscopic aggregation morphology in water solution of polymer and gels after flowing through cores with different permeabilities (×200).

In order to verify why the properties of the three systems change after flowing through the sand-packed cores, the atomic force microscopy was used to further investigate the molecular aggregation morphology of the three systems before and after flowing through the sand-packed cores [20,22]. Figure 6 shows that the stock solutions of the three systems have different molecular aggregation states than the solutions after flowing through the core.

(1) The stock solution of the polymer shows a tight molecular aggregation state before flowing through the sand-packed core. After flowing through the 8.4 D core, the polymer molecules still exhibit aggregation state, but the solution becomes thin, which also explains the reason for the decrease in molecular coil size and viscoelasticity of polymer solution. After flowing through a 1.0 D core, the molecular structure of the polymer is further destroyed, the degree of intermolecular aggregation is further reduced, but the solution is still in a continuous state, and there is still an interwound active force between molecules, so the solution still has a certain viscoelasticity to improve the sweep efficiency in deep formation.
(2) Before flowing through the sand-packed core, the phenolic gel shows a denser network aggregate than the polymer solution, and its viscoelasticity and molecular coil size are larger than those of the stock solution of polymer because it is a weak gel formed by chemical crosslinking of molecules, having a stronger intermolecular interaction. After flowing through the 8.5 D core, the intermolecular network structure is partially destroyed, and the intermolecular interaction becomes poor; however, the gel still shows a network aggregation morphology as a whole, so its viscoelasticity and molecular coil size are still higher than those of the polymer solution. After flowing through 1.0 D core, the phenolic gel, with large monomolecular diameter, is sheared into gel particles, so the network aggregate cannot be formed, and the viscoelasticity is significantly reduced, making the gel’s deep migration ability reduce correspondingly.
(3) Before flowing through the sand-packed cores, the aluminum gel is a weak gel formed by crosslinking of coordination bonds, so the intermolecular interaction is also strengthened, the network aggregates formed are dense, and the viscoelasticity and molecular coil size are higher than those of the stock solution of polymer. After flowing through 8.4 D core, because the monomolecular activity of the aluminum gel is weaker than that of the phenolic gel, its intermolecular network structure is more seriously destroyed than the phenolic gel, making the molecules of the aluminum gel impossible to form network aggregates, so the viscoelasticity and molecular coil size of the aluminum gel are significantly reduced. After flowing through 1.0 D core, the molecular structure of the aluminum gel is further broken, and the viscoelasticity is greatly reduced, reducing the gel’s deep migration ability.

### 3.4. Matching of Polymer and Weak Gel in Porous Media

In order to investigate the ability of the three systems to improve the mobility ratio and reduce reservoir permeability, the resistance factor and residual resistance factor of the three systems in cores with different permeabilities were measured for clarifying the matching of the polymer and weak gel in porous media [23,24].

**Figure 7:**
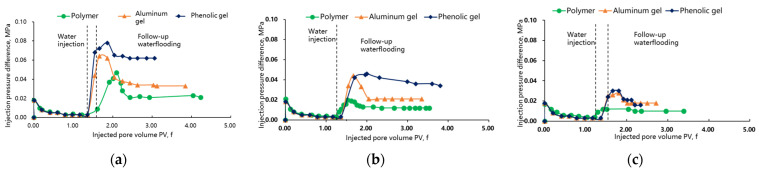
Changes of injection pressure of polymer and gel systems in cores with different permeabilities. (**a**) 0.09–0.45 D; (**b**) 0.95–5.1 D; (**c**) 1.6–8.1 D.

**Figure 8:**
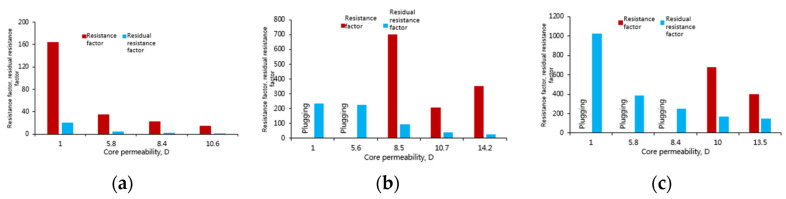
Resistance factor and residual resistance factor of polymer and gel systems in sand-packed cores. (**a**) Polymer; (**b**) Phenolic gel; (**c**) Aluminum gel.

Figure 7 shows roughly consistent variation in pressure difference for cores with different permeabilities: in the water injection stage, the pressure difference is small; after chemical injection, the pressure difference gradually increases, and the resistance factor also increases slowly; after gelling, the pressure increases sharply. Both aluminum gel and phenolic gel in Figure 7 show this phenomenon. The polymer exhibits less pressure increase than gels, mainly because the polymer has strong monomolecular activity, making it less capable of plugging the throats in porous media than gels. Given the same injection system, polymer, aluminum gel and phenolic gel are all characterized by lower injection pressure in case of higher permeability. Given the same permeability difference, phenolic gel corresponds to the highest injection pressure, followed by aluminum gel and polymer solution. This phenomenon is also observed in the resistance factor (Figure 8), and it is more obvious in case of decreasing permeability with constant permeability difference. Weak gel especially demonstrates lower monomolecular activity than the polymer, and exhibits a high resistance factor in cores with low permeability; thus, it is greatly affected by throat, leading to a huge loss in molecular coil size and viscoelasticity. In other words, a high resistance factor does not necessarily mean the best profile control and oil displacement effect.

It is found that polymer solution migrates in the cores with different permeabilities without any plugging. Thus, the polymer is capable of deep profile control and oil displacement in cores with permeability from 1 D to 10.6 D; the lower the permeability, the greater the resistance factor that the polymer can produce, and the stronger the sweep efficiency. However, when the permeability is higher than 8.4 D, the resistance factor is low, and the deep profile control capability becomes weak. The phenolic gel can create higher resistance factor than the polymer in the core with permeability higher than 8.5 D and does not block the core, so the phenolic gel is suitable for deep profile control and oil displacement in reservoirs with permeability ˃8.5 D. When the permeability is reduced to 5.8 D, the injection of phenolic gel creates core plugging, so for such reservoirs, phenolic gel can only produce profile control effect near wellbore, but not interfere deep formations. Aluminum gel is similar to phenolic gel, but its monomolecular activity after crosslinking is weaker. Therefore, aluminum gel is suitable for deep profile control and oil displacement in cores with permeability ˃10 D. For cores with permeability <10 D, aluminum gel can only work near wellbore, but cannot migrate to deep formations for profile control and oil displacement.

### 3.5. Profile Control and Oil Displacement Capability of Polymer and Weak Gel

**Figure 9:**
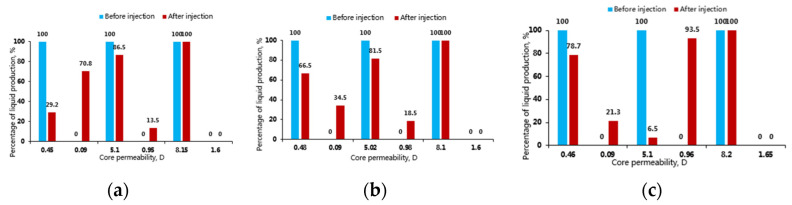
Percentage of liquid production of sand-packed cores in total liquid production before and after injection of the three systems. (**a**) Polymer; (**b**) Aluminum gel; (**c**) Phenolic gel.

**Figure 10:**
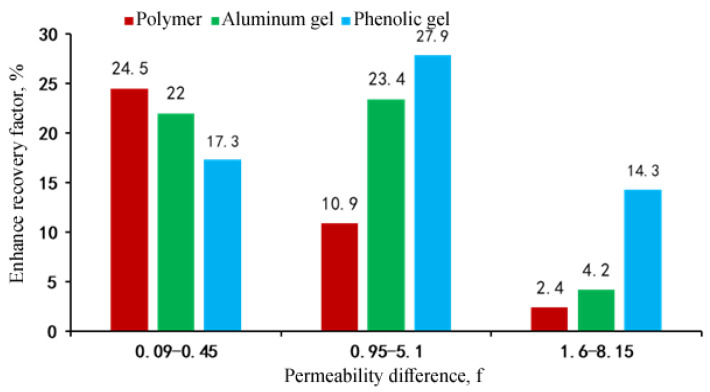
Comparison of enhanced recovery factor of polymer and gel systems.

**Figure 11:**
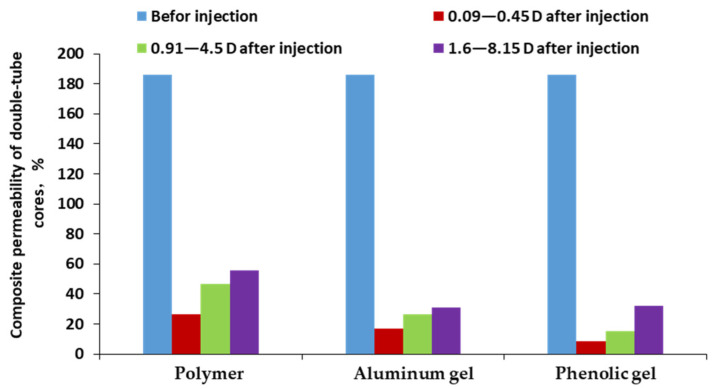
Comparison of composite permeability variation of polymer and gel systems.

Under the condition of 5 times of permeability difference of double-tube parallel sand-packed core, the adaptability of the three systems to the reservoir permeability difference of 0.09–0.45 D, 1–5.1 D and 1.6–8.1 D was investigated to test their oil displacement efficiencies and fluid diversion abilities. The experimental results (Figure 9 and Figure 10) show that the oil displacement efficiencies and fluid diversion abilities of the three systems have great differences in different permeability ranges. When the permeability ranges from 0.09 D to 0.50 D, the oil displacement efficiency of the polymer solution is the best, followed by aluminum gel and phenolic gel in turn, and the fluid diversion ability of the polymer solution is the strongest, with an enhanced recovery factor of 24.5%, which mainly attributes to the best fluidity of the polymer under lower permeabilities [24]. When the permeability ranges from 1.0 D to 5.1 D, the oil displacement efficiency of the phenolic gel is the best, followed by aluminum gel and polymer solution in turn, and the fluid diversion ability of the phenolic gel is the strongest, with an enhanced recovery factor of 27.9%. However, the polymer solution does not have fluid diversion ability; this is because when the permeability is higher than 1.0 D, the injection performance of polymer weak gel becomes better, and it also plays a role in deep profile control and oil displacement. When the permeability ranges from 1.6 D to 8.2 D, the oil displacement efficiency of phenolic gel is the best, followed by aluminum gel and polymer solution in turn (Figure 10), and all three systems do not have fluid diversion abilities; therefore, stronger plugging and profile control systems are needed in practical application. Figure 11 is the composite permeability contrast of the three systems before and after injection, showing that the overall core permeability has changed significantly before and after injection of the three systems. Under the condition of the same injection system, the lower the permeability, the greater the change value is. Under the condition of different injection systems, the change value of phenolic gel is the largest, followed by aluminum gel and polymer solution in turn, intuitively showing that the plugging strength of phenolic gel is the greatest, and that of the polymer solution is the weakest.

## 4. Conclusions

The molecular coil size, viscoelastic modulus and microscopic aggregation morphology in the water solution of three systems before and after core shearing were investigated, and the injection performance and profile control and oil displacement effect of the three systems in cores with different permeabilities were tested by physical simulation experiments.

(1) Polymer solution does not create intermolecular crosslinking and only forms intermolecular winding, with good flexibility of the molecular chain and strong monomolecular activity. Although the molecular coil size and viscoelasticity of polymer suffers a certain loss when it flows through cores, they tend to be stable when the permeability is lowered to a certain level. Therefore, the polymer solution is capable of deep profile control and oil displacement for reservoirs with permeability of 1–10 D. However, when the permeability is higher than 8.2 D, the resistance factor produced by the polymer becomes lower, making the capability of the polymer for deep profile control and oil displacement weaker.
(2) Phenolic gel and aluminum gel are weak gels formed by intermolecular chemical and physical crosslinking, so their molecular coil size and viscoelasticity are apparently greater than the polymer solution. They are highly capable of deep profile control and oil displacement in cores with permeability ˃8.5 D, and more capable than polymer solution. Since the monomolecular activity of weak gels is lower than that of the polymer, in cores with low permeability, the weak gel systems are more susceptible to shearing action; they may plug the cores during injection, meaning that their molecular coil size and viscoelasticity are seriously lost. Therefore, the two weak gel systems can only work for profile control near wellbore but cannot interfere with deep formations.

## Data Availability

Data available on request from the authors.

## References

[1] Li J., Qu Z., Zhao B. (1999). Study on the relationship among oil displacement efficiency and variation coefficient, rush coefficient and differential of permeability in Sanjianfang reservoir of the Shanshan oilfield. J. Xi’an Eng. Univ..

[2] Wang D., Cheng J., Wu J., Wang G. (2005). Application of polymer flooding technology in Daqing Oilfield. Acta Pet. Sin..

[3] Hou J., Guo L., Yuan F., Du Q., Yu B. (2008). Quantitative characterization for production performance of polymer flooding in different types of reservoirs in Shengli Oilfield. Acta Pet. Sin..

[4] Liu L., Li H. (2011). Numerical simulation of the permeability variation coefficient effect on polymer flooding. Oilfield Chem..

[5] Smith J.E. Performance of 18 polymers in aluminium citrate colloidal dispersion gels. Proceedings of the SPE International Symposium on Oilfield Chemistry.

[6] Sydansk R.D., Southwell G.P. (2000). More than 12 years’ experience with a successful conformance-control polymer-gel technology. SPE Prod. Facil..

[7] Smith J.E., Liu H., Guo S. Laboratory studies of in-depth colloidal dispersion gel technology for Daqing oil field. Proceedings of the SPE/AAPG Western Regional Meetings.

[8] Lei G., Li L., Nasr-El-Din H.A. (2011). New gel aggregates to improve sweep efficiency during waterflooding. SPE Reserv. Eval. Eng..

[9] Zhang Z., Zhao L., Cao B. (2018). Applicability evaluation and application of hydrophobic association polymer weak gel. Petrochem. Ind. Appl..

[10] Zhong W., Zhao B., Han S. (2020). Performance evaluation and application of deep profile control system in high temperature and high salinity reservoirs. Oilfield Chem..

[11] Zaitoun A., Makakou P., Blin N., Al-Maamari R.S., Al-Hashmi A.R., Abdel-Goad M., Al-Sharji H.H. (2012). Shear stability of EOR polymers. SPE J..

[12] Al Hashmi A.R., Al Maamari R.S., Al Shabibi I.S., Mansoor A.M., Zaitoun A., Al Sharji H.H. (2013). Rheology and mechanical degradation of high-molecular-weight partially hydrolyzed polyacrylamide during flow through capillaries. J. Pet. Sci. Eng..

[13] Lin M., Xin J., Li M., Dong Z. (2008). Study on shear stability of low concentration partially hydrolyzed polyacrylamide/aluminum citrate crosslinking system. Acta Polym. Sin..

[14] Zhang K. (1981). Polymeric Physics.

[15] Han P. (2014). Variation law of seepage field and oil displacement effect of alternate injection polymer flooding. Pet. Geol. Oilfield Dev. Daqing.

[16] Liu Z., Li Y., Gao W., Xue X., Wang S. (2017). Experimental study on variable resistance seepage law of polymer in vertical heterogeneous reservoirs. Xinjiang Pet. Geol..

[17] Zhang N., Lu X., Xie K. (2020). Seepage characteristics and influential factors of polymer weak gel under high salt and medium-low permeability conditions. Oilfield Chem..

[18] Liu Z., Cheng H., Xu C., Chen Y., Chen Y., Li Y. Effect of lithologyon pore-scale residual oil displacement in chemical flooding usingnuclear magnetic resonance experiments. Proceedings of the SPE EOR Conference at Oil and Gas West Asia.

[19] Zhao S., Pu W., Li K. (2019). Study on profile control ability of polymer microspheres to heterogeneous formation. Pet. Reserv. Eval. Dev..

[20] Li J., Liu Y., Gao Y., Cheng B., Fanle M.E.N.G., Huaimin X.U. (2018). Effects of microscopic pore structure heterogeneity on the distribution and morphology of remaining oil. Pet. Explor. Dev..

[21] Li J., Liu Y., Gao Y., Cheng B.Y., Jiang H.Q. (2019). Pore-scale study of the pressure-sensitive effect of sandstone and its influence on multiphase flows. Pet. Sci..

[22] Liu J., Lu X., Liu J., Shuqiong H.U. (2013). Mechanism and gelling effects of linked polymer solution in the core. Pet. Explor. Dev..

[23] Luo X.R., Zhao B., Ren X.J. (2021). Effect of mechanical properties of pre-crosslinked gel particles on micro migration and plugging. Lithol. Reserv..

[24] Sun Z. (2017). Research on the Evaluation Method of Polymer Microsphere Reservoir Adaptability and the Profile Control and Displacement Mechanism.

